# Molecular Diversity of HIV-1 among People Who Inject Drugs in Kuala Lumpur, Malaysia: Massive Expansion of Circulating Recombinant Form (CRF) 33_01B and Emergence of Multiple Unique Recombinant Clusters

**DOI:** 10.1371/journal.pone.0062560

**Published:** 2013-05-07

**Authors:** Wei Zhen Chow, Lai Yee Ong, Siti Humaira Razak, Yeat Mei Lee, Kim Tien Ng, Yean Kong Yong, Azureen Azmel, Yutaka Takebe, Haider Abdulrazzaq Abed Al-Darraji, Adeeba Kamarulzaman, Kok Keng Tee

**Affiliations:** 1 Centre of Excellence for Research in AIDS (CERiA), Department of Medicine, Faculty of Medicine, University of Malaya, Kuala Lumpur, Malaysia; 2 AIDS Research Center, National Institute of Infectious Diseases, Toyama, Shinjuku-ku, Tokyo, Japan; Institut Pasteur of Shanghai, Chinese Academy of Sciences, China

## Abstract

Since the discovery of HIV-1 circulating recombinant form (CRF) 33_01B in Malaysia in the early 2000 s, continuous genetic diversification and active recombination involving CRF33_01B and other circulating genotypes in the region including CRF01_AE and subtype B′ of Thai origin, have led to the emergence of novel CRFs and unique recombinant forms. The history and magnitude of CRF33_01B transmission among various risk groups including people who inject drugs (PWID) however have not been investigated despite the high epidemiological impact of CRF33_01B in the region. We update the most recent molecular epidemiology of HIV-1 among PWIDs recruited in Malaysia between 2010 and 2011 by population sequencing and phylogenetic analysis of 128 *gag-pol* sequences. HIV-1 CRF33_01B was circulating among 71% of PWIDs whilst a lower prevalence of other previously dominant HIV-1 genotypes [subtype B′ (11%) and CRF01_AE (5%)] and CRF01_AE/B′ unique recombinants (13%) were detected, indicating a significant shift in genotype replacement in this population. Three clusters of CRF01_AE/B′ recombinants displaying divergent yet phylogenetically-related mosaic genomes to CRF33_01B were identified and characterized, suggestive of an abrupt emergence of multiple novel CRF clades. Using rigorous maximum likelihood approach and the Bayesian Markov chain Monte Carlo (MCMC) sampling of CRF33_01B_pol_ sequences to elucidate the past population dynamics, we found that the founder lineages of CRF33_01B were likely to have first emerged among PWIDs in the early 1990 s before spreading exponentially to various high and low-risk populations (including children who acquired infections from their mothers) and later on became endemic around the early 2000 s. Taken together, our findings provide notable genetic evidence indicating the widespread expansion of CRF33_01B among PWIDs and into the general population. The emergence of numerous previously unknown recombinant clades highlights the escalating genetic complexity of HIV-1 in the Southeast Asian region.

## Introduction

By the end of 2011, the Joint United Nations Program on HIV/AIDS (UNAIDS) estimated around 4 million people were living with HIV in Southeast Asia, indicating an 8% increase compared to that in a decade ago. Furthermore, 280,000 new HIV infections had occurred within the same year [1], highlighting the increasing burden of HIV/AIDS faced by many countries in the region. Use of unsterile injecting equipment represents one of the most prevalent mode of HIV transmission as depicted in a 2008 study which estimated a total of 16 million people who injected drugs, of whom 3 million people were infected with HIV [2]. Transmission of HIV among people who inject drugs (PWID) continues to prevail in Southeast Asia, Eastern Europe and Latin America – at present, five countries are thought to be facing mega-epidemics from injecting drug use, namely China, Vietnam, Russia, Ukraine and Malaysia (where the term mega-epidemic refers to a country which has more than 75,000 registered cases of HIV infections in a particular risk group) [3]. In particular, Southeast and East Asia are home to around 4 million PWIDs in 2010 and displayed a HIV prevalence rate of almost 20% [4]. In Malaysia, a total of 94,841 cases of HIV infections have been reported by the end of 2011 since the country’s first confirmed case in 1986. The HIV epidemic is largely concentrated amongst PWIDs contributing to 70–80% of the overall HIV prevalence in the country [5].

In Southeast Asia, Thailand has been the epicenter of the HIV/AIDS epidemic for many decades. In this country, the early HIV epidemic was initiated by two HIV-1 genotypes, circulating recombinant form (CRF) 01_AE (CRF01_AE) and subtype B (including subtype B′, the Thai variant of subtype B), albeit circulating independently among different risk groups. HIV-1 CRF01_AE, believed to be of central African origin, initially propagated among commercial sex workers and their clients while subtype B′ circulated among PWIDs [6]. However, nearly a decade later in the mid-1990 s, CRF01_AE was reported to have infiltrated the PWIDs population, circulating in approximately 80% of PWIDs in Thailand [7]. As a result of the high CRF01_AE prevalence and the fact that drug trafficking activities were common across the region [8], CRF01_AE was later found to be disseminating widely among PWIDs in other parts of Southeast Asia and East Asia, including Cambodia, Vietnam, Malaysia, China, Taiwan, Korea and Japan [9]–[11]. To date, CRF01_AE is still circulating widely in Southeast Asia and causes the majority of HIV infections in the region [6], [12]. In Malaysia, the early HIV epidemic in the 1990 s implied a similar trend to that of Thailand’s initial HIV epidemic – CRF01_AE and subtype B′ were predominant among those with high-risk heterosexual exposures and injecting drug practices, respectively [13]–[15]. In the early 2000 s, Tee et al. reported that whilst subtype B′ remained the predominant subtype amongst PWIDs, the high genetic plasticity and co-circulation of distinct HIV-1 genotypes had contributed to the emergence of unique CRF01_AE/B′ inter-subtype recombinants in the population of PWIDs [16], [17], similar to other studies by Tovanabutra et al. reporting the upsurge of CRF01_AE/B′ recombinants in more than half of a high-risk cohort in Thailand [18].

Interestingly, co-circulation of and dual infection involving CRF01_AE and subtype B′ in Southeast Asia paved the way to the emergence of six novel and genetically distinct CRF01_AE/B′ recombinants of epidemiological significance in the region: CRF15_01B [19] and CRF34_01B [20] in Thailand, CRF33_01B [21] and CRF48_01B [22] in Malaysia, CRF51_01B [23] in Singapore, and CRF52_01B [24] both in Thailand and Malaysia. The emergence and distribution of these new CRFs is however not limited to the country of origin. For instance, CRF33_01B was disseminating widely in various high-risk populations in Malaysia, primarily in PWIDs [21]. In recent years, CRF33_01B has been steadily expanding across the borders to Singapore [25], [26], Indonesia [27], [28] and also Hong Kong [29]. In addition to the spread of CRF33_01B into neighbouring countries, continuous diversification through active recombination between CRF33_01B and other CRF01_AE and/or subtype B′ strains have resulted in the upsurge of new CRF and unique recombinant forms (URF) that show divergent yet phylogenetically-related mosaic genomes with CRF33_01B [22], [27], [30]. Therefore, co-circulation of viral lineages, continual gene flow between and among the concentrated and generalized host populations and frequent genetic recombination – involving various established CRF01_AE and subtype B′ clades, the recently discovered CRFs, and unique CRF01_AE/B′ recombinants – would most likely lead to the escalation of HIV-1 genetic complexity in the region. Although unsafe injection of unsterile equipment remain the major mode of HIV transmission in Malaysia and also elsewhere in the region, recent data on the genetic dispersion of various HIV-1 genotypes among PWIDs, including the history of transmission and dominance of distinct CRFs, is unavailable. Therefore, in this study, we aim to investigate the rapidly evolving molecular epidemiological profiles of HIV-1 among PWIDs in Kuala Lumpur, Malaysia through the incorporation of rigorous phylogenetic analyses and a high-resolution Bayesian resampling approach.

## Materials and Methods

### Ethics Statement

The study was approved by the University Malaya Medical Centre (UMMC) Medical Ethics Committee. Standard, multilingual consent forms allowed by the Medical Ethics Committee were used. Written consent was obtained from all study participants.

### Study Subjects and Specimens

A total of 158 consented PWIDs were recruited among inmates of a prison (n = 118) and attendees of a needle syringe exchange program (n = 40) in Kuala Lumpur, Malaysia between the period of July and November 2010 and April and May 2011, respectively. All recruited subjects reported a history of unsafe injecting drug use in the questionnaires provided. Plasma samples were collected from all 158 subjects, serologically determined to be HIV-1 positive and stored at −80°C until processed. No HIV-2 infections were detected.

### Viral RNA Isolation, Amplification and Sequencing

HIV-1 Viral RNA was extracted from plasma by column purification method (QIAamp Viral RNA Mini Kit, Qiagen, Hilden, Germany) and reverse transcribed into cDNA using SuperScript III RNase H^−^ Reverse Transcriptase (Invitrogen, Carlsbad, California, USA) and random hexamers (Applied Biosystems, USA) according to the manufacturer’s instructions. Nested PCR was performed separately to amplify the *gag-pol* gene that spans the *gag* (p24), protease (PR) and reverse transcriptase (RT) genes using QIAGEN HotStarTaq *Plus* DNA polymerase (Qiagen, Hilden, Germany). The thermal conditions used in the first and second round of PCR were initial denaturation at 95°C for 5 minutes, followed by denaturation at 94°C for 30 seconds, annealing at 50°C for 30 seconds, elongation at 72°C for 1 minute and 30 seconds and a final elongation step at 72°C for 10 minutes. The primers [31] used to amplify the *gag*-PR genes in the first round of PCR were 507A (5′-AAG GAA CCC TTT AGA GAC TAT GTA GA-3′; nucleotide positions relative to HXB2∶1657–1682) and 503B (5′-TAT GGA TTT TCA GGC CCA ATT TTT G-3′; HXB2∶2692–2716), followed by primers 508A (5′-GTA AAA AAT TGG ATG ACA GAA ACC TTG-3′; HXB2∶1726–1752) and 504B (5′-ACT TTT GGG CCA TCC ATT CC-3′; HXB2∶2611–2592) in the second round of PCR. Meanwhile for RT gene amplification, primers 325A (5′-GGA AAC CAA AAA TGA TAG GGG GAA TTG GAG G-3′; HXB2∶2377–2407) and 326B (5′-CTG TAC TTC TGC TAC TAA GTC TTT TGA TGG G-3′; HXB2∶3539–3509) were used in the first round of PCR while primers 327A (5′-GTG GAA AAA AGG CTA TAG GTA CAG-3′; HXB2∶2452–2475) and 328B (5′-CTG CCA ACT CTA ATT CTG CTT C-3′; HXB2∶3462–3441) were used in the second round of PCR. The PCR products were purified and population sequencing was performed in an ABI PRISM 3730XL DNA Analyzer using BigDye terminators (Applied Biosystems, Foster City, California, USA).

### Phylogenetic and Recombination Analysis

The *gag-pol* nucleotide sequences were assembled using DNASis Max (Hitachi, Japan) to form a 1683 bp contig (HXB2∶1753–3440) for samples in which both *gag*-PR and RT genes were successfully amplified. The *gag-pol* contigs were aligned with the HIV-1 reference subtypes and CRFs downloaded from the Los Alamos HIV database (http://www.hiv.lanl.gov/) using ClustalX 2.0. The nucleotide sequences were then manually adjusted using BioEdit 7.0 with reference to the HIV Sequence Compendium 2011 (http://www.hiv.lanl.gov/) to ensure accurate codon alignment. Phylogenetic trees were constructed by the neighbour-joining method [32] based on the Kimura two-parameter model with a transition-transversion ratio of 2.0 [33] implemented in MEGA 5.05 [34]. The reliability of the branching orders were analysed by bootstrap analysis of 1000 replicates. For samples in which either *gag*-PR or RT was successfully amplified, the nucleotide sequences were manually aligned and used to construct phylogenetic trees for the *gag*-PR (HXB2∶1753–2591, 834 bp) or RT genes (HXB2∶2476–3440, 966 bp) respectively in order to determine the evolutionary relationship. Nucleotide sequences obtained in the study were screened for novel HIV-1 recombinant structures using the Recombinant Identification Program (RIP) available at the Los Alamos HIV database. Following RIP estimation, bootscanning and informative-site analyses [35] were performed on recombinant genotypes using SimPlot version 3.5.1 [36]. In order to confirm the putative parental origin of each recombinant segment, sub-region neighbour-joining tree analyses were constructed in MEGA 5.05. In this study, we defined a “cluster" in the context of recombination as a clade consisting of at least three sequences [37]–[39] displaying identical recombination structures and breakpoints in the *gag-pol* genes. All sequences reported in this study have been deposited in GenBank with accession numbers KC477938 to KC478065.

### Estimating the Past Population Dynamics of CRF33_01B

In order to infer the evolutionary history and population dynamics of HIV-1 CRF33_01B in Kuala Lumpur and also other regions in Malaysia, all previously reported genetic sequences isolated from various risk populations were retrieved from the Los Alamos HIV database and our in-house laboratory database, including the *gag-pol* gene sequences newly amplified from all CRF33_01B isolates reported in this study. As a result, a total of 242 CRF33_01B sequence data set with recorded sampling year spanning 2005–2012 (inclusive of 152 and 90 sequences with known and unknown transmission risks, respectively) sampled from the central, northern and eastern states in Malaysia were analysed. All CRF33_01B sequences were aligned and maximum likelihood analysis of the 152 sequences with known transmission risks was performed using PAUP* v4.0 beta [40]. Using the comprehensive alignment comprising of 242 1 kb *pol* gene sequences of CRF33_01B genome (HXB2∶2253–3260), the past population dynamics of CRF33_01B was estimated using the coalescent-based Bayesian Markov chain Monte Carlo (MCMC) sampling method performed in BEAST v1.7 [41]. An uncorrelated lognormal relaxed molecular clock [42] was used for the Bayesian skyline reconstructions. The evolutionary rates and tree topologies were analysed using the general time-reversible (GTR) [43] nucleotide substitution parameter with gamma distributed among-site rate variation with four rate categories (γ_4_) [44]. Each MCMC chain was run for 30 million states with a sampling rate of every 10,000 states. The MCMC output was checked for convergence and effective sampling size using Tracer v1.4 (available at http://tree.bio.ed.ac.uk) with 10% of each chain discarded as burn-in.

## Results

### Spread of HIV-1 CRF33_01B and Emergence of Multiple Recombinant Clusters among PWIDs in Kuala Lumpur, Malaysia

The recent HIV-1 genotype distribution among PWIDs was assessed among consented subjects recruited from a prison and a needle syringe exchange program in Kuala Lumpur, Malaysia. The HIV-infected adult subjects included 138 males with an age range of 23–56 years old (mean: 36.5±9.6 years old) and 20 females with an age range of 24–45 years old (mean: 35.5±6.8 years old) with a mean CD4^+^ T cell count of 438 cells/µl. Among 116 subjects with available serological data, approximately 93% were anti-HCV positive. The majority (89.8%) of subjects were antiretroviral (ARV)-naive at the time of sample collection. HIV-1 genotype assignment was determined by population sequencing and phylogenetic analysis of the *gag-pol* gene spanning the *gag-*PR and RT regions. From 158 HIV-positive PWIDs, the *gag-pol* genes were successfully amplified in 81% (128/158) of the study subjects in which a 1683 bp *gag-pol* contig (HXB2∶1753–3440) was assembled in 77% (98/128) while partial *gag-*PR (HXB2∶1753–2591, 834 bp) or RT (HXB2∶2476–3440, 966 bp) genes were sequenced in 13% (16/128) and 11% (14/128), respectively of the population.

**Figure 1:**
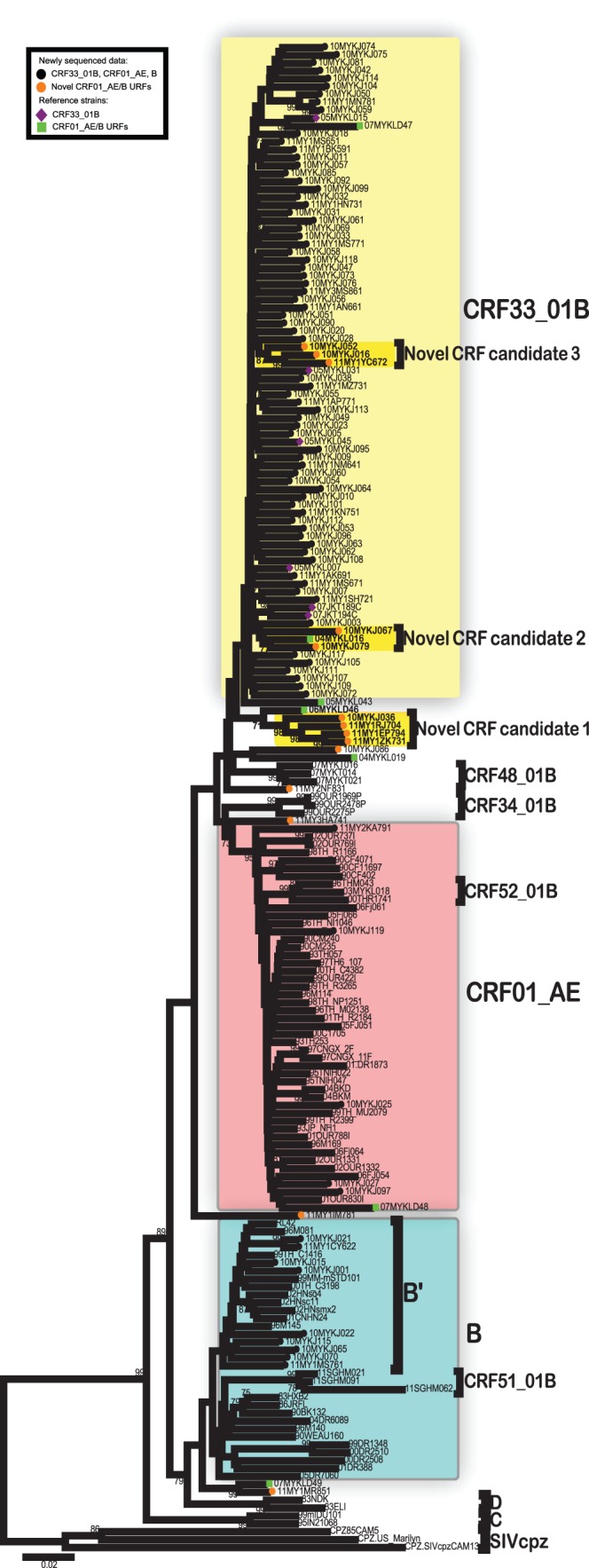
Phylogenetic analysis of the 1.6 kb HIV-1 *gag-pol* sequences (HXB2∶1753–3440) amplified among people who inject drugs (PWID) in Kuala Lumpur, Malaysia. The *gag-pol* genes (n = 98) were sequenced, codon-aligned and manually adjusted with the HIV-1 reference subtypes and circulating recombinant forms (CRFs) retrieved from the Los Alamos HIV database (http://www.hiv.lanl.gov/). Neighbour-joining tree was constructed in MEGA 5.05 [34] using Kimura 2-parameter method of nucleotide substitutions to estimate pair-wise evolutionary distance and the reliability of the branching nodes were assessed by bootstrap analysis of 1000 replicates. Reference strains of established and informative HIV-1 genotypes – CRF01_AE, subtype B (including Thai B′), CRF15_01B, CRF34_01B CRF33_01B, CRF48_01B, CRF51_01B, CRF52_01B and other relevant unique recombinant forms (URFs) circulating in Southeast Asia were also included in the analysis to improve tree resolution. Other HIV-1 genotypes – subtypes C, D and SIVcpz reference strains (CPZ.US_MARILYN and CPZ.SIVcpzCAM13) were included as outgroup. The newly emerged unique recombinant clusters identified in this study were highlighted as CRF candidate 1 (10MYKJ036, 11MY1RJ704, 11MY1ZK731 and 11MY1EP794), CRF candidate 2 (10MYKJ067, 10MYKJ079 and 04MYKL016) and CRF candidate 3 (10MYKJ016, 10MYKJ052, 11MY1YC672 and 11MY1JJ741). Bootstrap values of greater than 70% were indicated on the branch nodes. The scale bar represents 2% genetic distance (0.02 substitutions per site).

Based on neighbour-joining phylogenetic reconstructions of the *gag-pol* genes (**Figure 1**) and other partial *gag-*PR and RT genes (**Figure S1a and S1b**), 71% (91/128) of PWIDs isolates were grouped with the HIV-1 CRF33_01B reference sequences from the database, indicating a high prevalence of CRF33_01B circulating in the study population. HIV-1 CRF01_AE/B recombinants were detected at 13% (16/128) (described in detail below). This is followed by a lower prevalence rate of other established HIV-1 genotypes circulating in the region – subtype B′ and CRF01_AE attributing to 11% (14/128) and 5% (7/128), respectively of the HIV-1 infections among PWIDs. Unlike HIV-1 subtype B′ of Thai origin, western lineages of subtype B were not identified among the PWIDs in this study. Likewise, other previously described CRFs in Southeast Asia such as CRF15_01B, CRF34_01B, CRF48_01B, CRF51_01B and CRF52_01B were also not detected in the study population.

**Figure 2:**
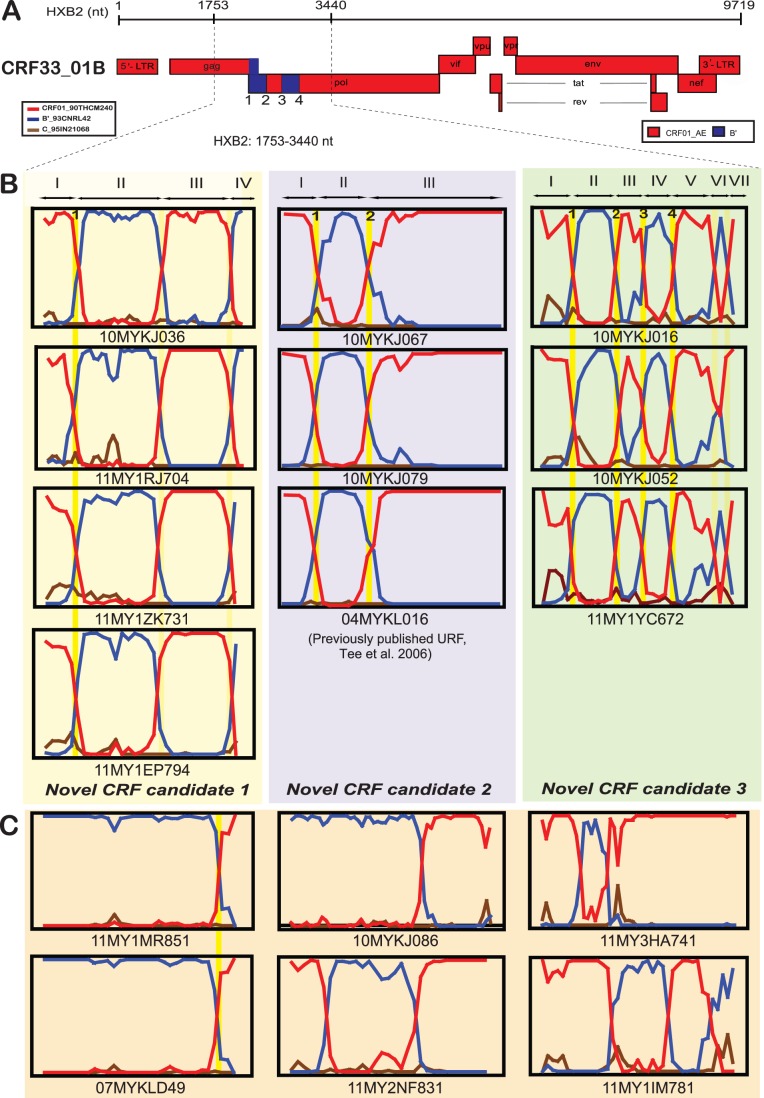
Bootscan analyses of HIV-1 unique recombinant forms (URFs) isolated from people who inject drugs (PWID) in Malaysia. **A**, Schematic representation of the full length recombinant structure of CRF33_01B with the four unique recombination breakpoints labelled as 1–4 (HXB2∶2053–2063 nt, 2375–2416 nt, 2538–2540 nt and 2841–2875 nt, respectively) in the *gag-pol* region [21]. **B**, Bootscanning plots of three novel CRF candidates (1 to 3) generated from the 1.6 kb *gag-pol* gene (HXB2∶1753–3440 nt) using SimPlot version 3.5.1 [36]. HIV-1 reference strains CRF01_CM240 (CRF01_AE) and B′_RL42 (Thai subtype B′) were selected as the putative parental genotypes by similarity plotting and C_95IN21068 (subtype C) as an outgroup, with a window size of 200 nucleotides moving along the alignment in increments of 50 nucleotides to define the recombination structures. The shared recombination breakpoints between the URFs were highlighted and in cases where the breakpoints were also shared with CRF33_01B, these breakpoints were further numbered (1 to 4). Each sub-region of these CRF candidates was also indicated and described further in **Figure 3**. **C,** Bootscanning plots of unique recombinant forms (URFs) –11MY1MR851 (sharing a similar recombination breakpoint with 07MYKLD49 [46]), 10MYKJ086, 11MY2NF831, 11MY3HA741 and 11MY1IM781 displaying distinct recombinant structures in the 1.6 kb *gag-pol* sequences amplified among the PWIDs study population. For clarity, bootscanning plots of partial RT and *gag-*PR sequences (11MY1JJ741 and 10MYKJ084, respectively) were not displayed here but their recombination structures have been discussed in detail in the text.

Of note, four isolates (10MYKJ036, 11MY1RJ704, 11MY1ZK731 and 11MY1EP794) showed unique tree topology by grouping together with a previously reported CRF01_AE/B recombinant (06MYKLD46) [45] at the base of the CRF33_01B clade, supported by a bootstrap value of 71% in the neighbour-joining tree (**Figure 1**). The four isolates sequenced in this study seemed to have diverged from 06MYKLD46 lineage and formed a well-supported clade (98% bootstrap value). Nucleotide sequences of these five isolates were submitted to the RIP tool at the HIV database and later bootscanned (using HIV-1 CRF01_CM240 and B′_RL42 as the putative parental genotypes) by SimPlot, showing evidence of identical CRF01_AE/B mosaic genome structure shared among the four isolates except 06MYKLD46 (**Figure 2B** **and Figure S2**). Informative sites analyses revealed the recombination of two subtype B′ segments at HXB2 positions 2064–2665 and 3260–3440 nt shared by all four isolates (10MYKJ036, 11MY1RJ704, 11MY1ZK731 and 11MY1EP794) in addition to the CRF01_AE segments at positions 1753–2052 and 2690–3194 nt. In all four isolates, similar multiple recombination breakpoints were identified at positions 2053–2063, 2666–2689 and 3195–3259 nt.

Furthermore at the base of subtype B or B′ clade, an isolate 11MY1MR851 formed a clade with a previously reported CRF01_AE/B recombinant (07MYKLD49) [46] and supported by 99% bootstrap value (**Figure 1**). Further analysis was performed to determine their putative recombination structures (**Figure 2C**) where it was revealed that both isolates shared similar recombination breakpoints with a subtype B′ fragment at position 1753–3164 nt and followed by a CRF01_AE segment at position 3218–3440 nt. On the other hand, there were four isolates that were not grouped with any known reference subtypes, CRFs or URF, namely 10MYKJ086, 11MY2NF831, 11MY3HA741 and 11MY1IM781 (**Figure 1**). Isolate 10MYKJ086 was located outside the CRF33_01B clade, displaying a mosaic genome pattern with subtype B′ and CRF01_AE segments at positions 1753–2840 and 2876–3440 nt, respectively (**Figure 2C**). Secondly, an isolate located at the base of CRF48_01B clade, 11MY2NF831 displayed a subtype B′ segment at position 2173–2665 nt and two CRF01_AE segments at positions 1753–2110 nt and 2735–3440 nt. Thirdly, isolate 11MY3HA741 located outside the CRF01_AE clade showed a subtype B′ segment at position 2168–2225 nt and two CRF01_AE segments at positions 1753–2135 and 2291–3440 nt. Finally, a more complex recombination structure was observed in isolate 11MY1IM781 whereby two subtype B′ segments were identified at positions 2426–2810 and 3161–3440 nt with CRF01_AE segments at positions 1753–2309 and 2876–3147 nt.

**Figure 3:**
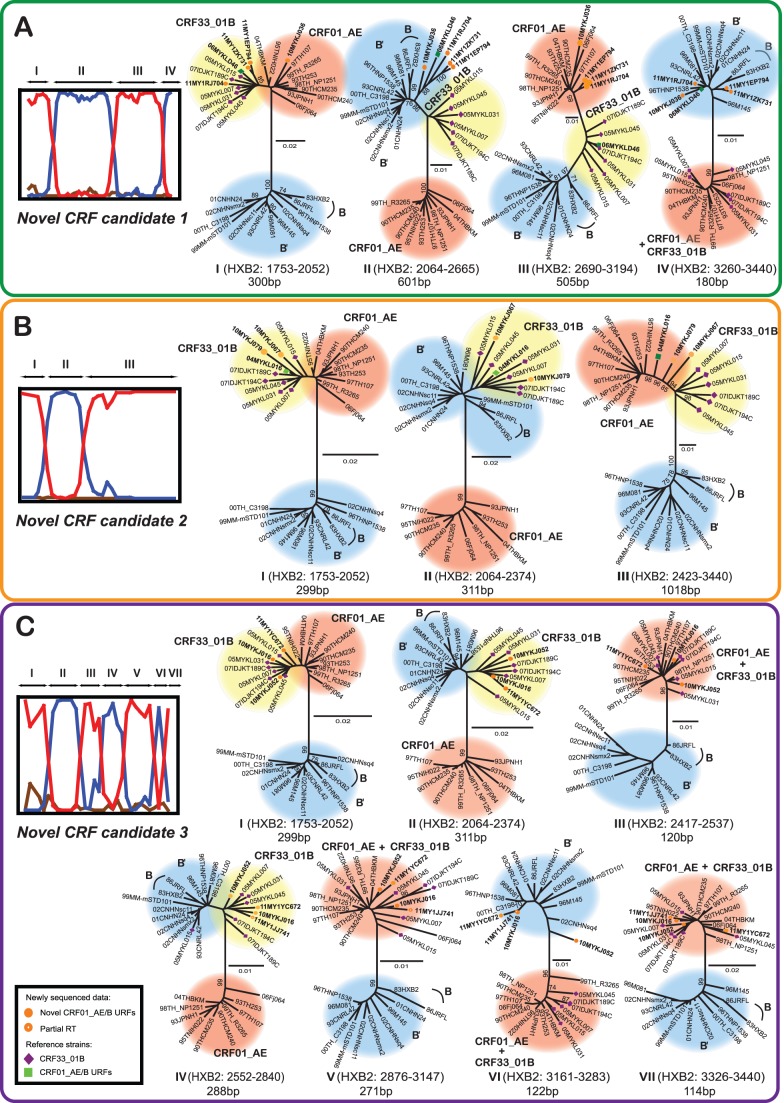
Sub-region trees analyses of the unique recombinant clusters. The *gag-pol* genes were sequenced, codon-aligned and manually adjusted with the HIV-1 reference subtypes and circulating recombinant forms (CRFs) retrieved from the Los Alamos HIV database (http://www.hiv.lanl.gov/). The 1.6kb *gag-pol* sequences of the unique recombinant clusters were subdivided into regions (denoted by Roman letters) in relation to the unique recombination breakpoints estimated by informative site analysis. Sub-region neighbour-joining trees were constructed in MEGA 5.05 [34] using Kimura 2-parameter method for nucleotide substitutions to estimate pair-wise evolutionary distance. The reliability of the branch nodes were assessed by bootstrap analysis of 1000 replicates. Bootscan plots and sub-region neighbour-joining trees for each CRF candidates were shown: **A**, novel CRF candidate 1 (10MYKJ036, 11MY1RJ704, 11MY1ZK731 and 11MY1EP794), **B**, novel CRF candidate 2 (10MYKJ067, 10MYKJ079 and 04MYKL016) and **C**, novel CRF candidate 3 (10MYKJ016, 10MYKJ052, 11MYIYC672 and 11MY1JJ741). HIV-1 reference strains CRF01_CM240 (CRF01_AE) and B′_RL42 (Thai subtype B′) were selected as the putative parental genotypes by similarity plotting and included in the bootscan plots (from **Figure 2**). The novel CRF candidate 1 or CRF33_01B were highlighted where the sequences formed a clade in the respective sub-region trees. In cases where the sub-region of CRF33_01B clustered with the reference strains of either putative parental genotypes - CRF01_AE or B′ showing the parental origin, the parental genotype was highlighted. The HXB2 nucleotide positions and sequence length of individual sub-regions were indicated in the illustration. Bootstrap values of greater than 70% were indicated on the branch nodes. The scale bar for respective sub-region trees was also indicated (in substitutions per site).

Given the significant presence of HIV-1 CRF01_AE/B recombinants detected in the *gag-pol* genes, we proceeded to screen every newly generated sequence data using a suite of recombination analysis tools (RIP, bootscan and informative sites analyses mentioned above) to identify the presence of other URFs that may not be readily identified based on the tree topology. Interestingly, based on the 1.6kb *gag-pol* genes, we identified two other unique lineages within the CRF33_01B clade that showed distinct genomic structures compared to that of CRF33_01B (**Figure 1**). A group of sequences consisting of isolates 10MYKJ067, 10MYKJ079 and a previously reported URF, 04MYKL016 [21] showed a short subtype B′ segment (311bp) identified at position 2064–2374 nt in relation to the unique recombination breakpoints at positions 2053–2063 and 2375–2422 nt shared by all three isolates. Meanwhile CRF01_AE segments were identified at positions 1753–2052 and 2423–3440 nt in these isolates. The origin of each segment was confirmed by sub-region tree analyses using a selected panel of CRF01_AE and B′ parental strains (**Figure 3**). It is important to note that the recombination pattern for these isolates was related to that of CRF33_01B, although the subtype B′ segment in CRF33_01B (at position 2541–2840 nt) [21] was not present among these novel recombinants (**Figure 2A and 2B**). In the other lineage, three isolates (10MYKJ016, 10MYKJ052 and 11MY1YC672) clustered with 87% bootstrap support also shared similar unique recombination breakpoints upon detailed recombination analyses. In these three isolates, three subtype B′ segments were identified at positions 2064–2374, 2552–2840 and 3161–3283 nt whereas four segments at positions 1753–2052, 2417–2537, 2876–3147 and 3326–3440 nt were identified as CRF01_AE (**Figure 2B**). Moreover, analysis of partial RT (HXB2∶2476–3440) sequences also revealed an additional isolate (11MY1JJ741) closely related to the isolates 10MYKJ016, 10MYKJ052 and 11MY1YC672 (supported by an 86% bootstrap value) and shared similar recombinant structures in the RT region as described (**Figure S1a** **and** **Figure 2B**). Likewise, analysis of the partial *gag*-PR sequences had identified an isolate within the CRF33_01B clade harbouring unique recombinant structures distinct from any other isolates. For example, partial *gag*-PR sequence of isolate 10MYKJ084 displayed a subtype B′ segment at position 2168–2374 nt and CRF01_AE segments at positions 1753–2135 and 2417–2591 nt.

Altogether, the present study identified a high prevalence of novel CRF01_AE/B recombinants displaying unique recombination structures in 13% (16/128) of PWIDs study population. This finding indicated the increasing genetic diversity of HIV-1 with a higher prevalence of recombinants (CRF33_01B and novel CRF01_AE/B recombinants) comprising a total of 84% of the HIV-1 infections among PWIDs.

### History and Transmission Pattern of HIV-1 CRF33_01B in Malaysia

HIV-1 CRF33_01B was first characterized in 2003 among various high-risk populations in Malaysia [17], [21]. The present study, deemed to be the first large-scale molecular epidemiological surveillance conducted among HIV-1 infected PWIDs in Kuala Lumpur, Malaysia, revealed a significantly high prevalence of CRF33_01B (71%) in this population, warranting an in-depth investigation into the history and transmission pattern of CRF33_01B extending to various risk groups in the country. Using a comprehensive data set of 242 CRF33_01B sequences (*pol* region, HXB2∶2253–3260) spanning year 2005–2012 among individuals from various risk groups (inclusive of 152 and 90 sequences with known and unknown transmission risks, respectively) and geographical origins (central, northern and eastern states of Malaysia), the past population dynamics of CRF33_01B was estimated by incorporating high-resolution analyses of maximum likelihood [40] and Bayesian sampling approach [47]. The data set of CRF33_01B_pol_ sequences were amplified from HIV-1 positive samples collected from the following regions: the central states of Malaysia (n = 142) including Kuala Lumpur (n = 54, including four CRF33_01B reference sequences [21]), Selangor (n = 86) and Negeri Sembilan (n = 2); the northern states (n = 85) including Kelantan (n = 79) and Perak (n = 6); and the eastern state (n = 13) of Pahang [22]. Indonesian CRF33_01B reference sequences (n = 2) were also retrieved from the online database [27] and included in the analyses.

**Figure 4:**
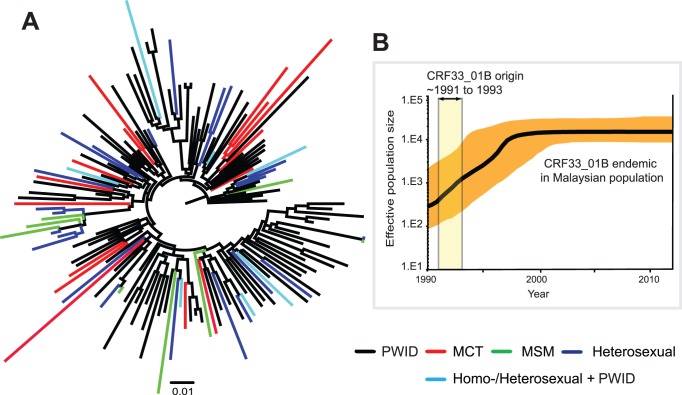
Maximum likelihood analysis and past population dynamics of HIV-1 CRF33_01B disseminating among various risk groups in Southeast Asia (Malaysia and Indonesia). The maximum likelihood reconstruction and past population dynamics of all available CRF33_01B isolates was estimated by PAUP* v4.0 beta [40] and the Bayesian sampling approach implemented in BEAST v1.7 [47], respectively. A total of 242 CRF33_01B sequences (*pol* region, HXB2∶2253–3260) spanning year 2005–2012 among individuals from various risk groups (inclusive of 152 and 90 sequences with known and unknown transmission risks, respectively) and geographical origins (central, northern and eastern states of Malaysia) and two full-length sequences from Indonesia were analyzed. The data set of CRF33_01B_pol_ sequences were amplified from HIV-1 positive samples collected from the following regions: the central states of Malaysia (n = 142) including Kuala Lumpur (n = 54, including four CRF33_01B reference sequences [21], Selangor (n = 86) and Negeri Sembilan (n = 2); the northern states (n = 85) including Kelantan (n = 79) and Perak (n = 6); and the eastern state (n = 13) of Pahang [22]. Indonesian CRF33_01B reference sequences (n = 2) were retrieved from the online database [27] and included in the analyses. **A**, Maximum likelihood analysis of CRF33_01B_pol_ data set containing 152 sequences with known transmission risks revealed a phylogenetic tree with star-like appearance, in which the terminal tree branches (mean length: 0.025±0.014) were in general longer than the internal branches (mean length: 0.005±0.005), possibly suggesting a high growth rate for CRF33_01B during the early phase of the epidemic [48]. In addition, tree topology showed the dissemination of HIV-1 CRF33_01B from PWIDs to other risk groups including the low-risk populations, notably among children who acquired infections through their mothers. **B**, The Bayesian skyline plot estimated the past population dynamics of CRF33_01B among various risk groups in Malaysia using the CRF33_01B_pol_ data set of 242 sequences mentioned above (and also in the text). The x-axis represents the time (in units of year) and y-axis represents the effective population size of CRF33_01B. The thick solid line in the plot represents the median estimate and the shaded region represents the 95% highest posterior density (HPD) credible region. The Bayesian skyline plot revealed an exponential increase in effective number of CRF33_01B infections in the host population since its reported emergence around 1991 to 1993 [31] until early 2000s and remained stable thereafter at a high prevalence, suggesting persistent viral transmission and endemicity of CRF33_01B among various risk groups in Malaysia.

Genealogy-based maximum likelihood analysis of CRF33_01B_pol_ data set containing 152 sequences with known transmission risks revealed a phylogenetic tree with star-like appearance in which the terminal tree branches (mean length: 0.025±0.014) were in general longer than the internal branches (mean length: 0.005±0.005) (**Figure 4A**). A number of CRF33_01B sub-lineages were observed, with isolates from the PWIDs predominantly sampled from the central states located at the base of these lineages showing apparent founder effect. Of note, internal nodes of CRF33_01B sequences from pediatric subjects who acquired infection from their mothers were positioned near the terminal or the tip of the tree. Next, in order to investigate the demographic history of CRF33_01B infections in Malaysia, specifically the past population dynamics, we estimated the effective population sizes of CRF33_01B through time by generating a Bayesian skyline plot using a total of 242 CRF33_01B_pol_ data set (inclusive of 90 sequences with unknown transmission risks) (**Figure 4B** **)**. From the plot, it was observed that the effective population size of CRF33_01B had remained significantly high since early 2000s for almost a decade, suggesting that the virus had established itself among various risk groups in the HIV-infected population along with other main circulating genotypes - CRF01_AE and subtype B′ in Malaysia. The plot correlated well with our current epidemiological finding reported in this study where CRF33_01B was massively expanding in the PWIDs study population.

## Discussion

People who inject drugs comprise 70% of the total HIV-1 infection cases reported in Malaysia from 1986–2011. Among an estimated 170,000 PWIDs in Malaysia by the end of 2011, approximately 65,032 cases of HIV-1 infections had been reported in the population and 14% (9,141) suffered AIDS-related deaths [5]. Previous molecular epidemiological studies indicated a high prevalence of HIV-1 CRF33_01B among PWIDs compared to other risk groups involving heterosexuals and homo−/bi-sexual males [21]. Similar investigation conducted among PWIDs in a remote fishing community from the eastern state of Malaysia reported similar findings [22]. However these studies were often limited in sampling size which hindered an in-depth HIV-1 molecular surveillance among PWIDs to be conducted in the country. In the present study, a large-scale molecular epidemiological surveillance and phylodynamic analysis of HIV-1 were conducted among 158 consented PWIDs recruited between 2010 and 2011 in Kuala Lumpur, Malaysia based on population sequencing and phylogenetic reconstructions of the HIV-1 *gag-pol* gene. We had for the first time provided robust genetic evidence highlighting the massive spread of HIV-1 CRF33_01B among the PWIDs, its epidemiological influence in other low-risk populations, and the evolutionary history of CRF33_01B prior to establishing its endemicity in the country.

In this study, approximately 71% of the recruited PWIDs population were found to be infected with HIV-1 CRF33_01B. The finding suggested that a profound epidemiological shift had occurred in the recent years following the first description of CRF33_01B almost a decade ago [17], [21]. Previously established HIV-1 genotypes – subtype B′ and CRF01_AE which played an important role during the early HIV-1 epidemic among PWIDs and heterosexuals, respectively in Southeast Asia including Malaysia [6], [9], [13]–[15] were reported at a substantially lower frequency with a combined prevalence of less than one-fifth of the total study population. Globally, 86% of HIV-1 infections in South and Southeast Asia (excluding India) is attributed to CRF01_AE while HIV-1 recombinants (including CRFs and URFs) comprised 20% of the infections worldwide [12]. Interestingly, CRF33_01B and multiple novel CRF01_AE/B′ unique recombinants identified in this study comprised an alarming 84% of total HIV-1 infections among PWIDs. The remarkably high prevalence of CRF33_01B and CRF01_AE/B′ recombinants described implied the escalating genetic diversity and complexity of HIV-1 in Malaysia compared to almost two decades ago when CRF01_AE and subtype B′ were the two major circulating strains reported among this population [13]–[15]. On the other hand, the western lineage of subtype B and other CRFs previously described in neighbouring countries (CRF15_01B and CRF34_01B from Thailand, CRF51_01B from Singapore and CRF52_01B from both Malaysia and Singapore) were not identified in the present study, suggesting the importation of foreign HIV-1 lineages into the injecting drug population in Malaysia was uncommon or a possible extinction of these imported lineages within the enclosed injecting drug networks in Malaysia. Viral population dynamics was estimated to illustrate the past changes in effective population size of CRF33_01B, which reportedly emerged between 1991 and 1993 [31]. Mapping back to almost two decades, the Bayesian skyline plot revealed an exponential increase in the effective number of CRF33_01B infections in the host population since its emergence until early 2000 s and remained stable thereafter at a high prevalence, suggesting persistent viral transmission and endemicity of CRF33_01B among various risk groups in Malaysia (**Figure 4B**). The star-like tree structure generated by maximum likelihood analysis, as indicated by terminal branches that were generally longer than the internal branches, was in concordance with the high growth rate of CRF33_01B during the early phase of the epidemic [48] (**Figure 4A**). Spatial analysis based on phylogenetic reconstruction showed vague evidence of directional gene flow from the central to other northern and eastern states, although a high proportion of early transmission events can be traced to PWIDs recruited from the central region (**Figure S3**). Furthermore, the dissemination of HIV-1 CRF33_01B from PWIDs to other risk groups including low-risk populations, most notably among children (mean age: 4.2 months) who acquired infections through their mothers via mother-to-child transmission, was also observed (**Figure 4A**). Together with CRF33_01B sequences isolated from pediatric subjects mainly recruited from the northern states [49], coalescent-based analysis showed considerable placement of the internal nodes for pediatric CRF33_01B lineages around the terminal positions of the tree, indicating a relatively recent divergence times. To our knowledge, the finding suggested for the first time the increasing transmission of HIV-1 CRF33_01B from PWIDs to the general population in the country. It is therefore not unexpected if more distinct inter-subtype recombinants involving CRF01_AE, subtype B′ and the recent highly prevalent CRF33_01B genotypes emerge encompassing various high and low risk populations infected with HIV-1, further increasing the genomic diversity of the complex HIV-1 molecular epidemiology in the general population.

In effect, multiple novel unique recombinants were identified in this study among 13% of PWIDs by analysing the 1.6 kb *gag-pol* and partial PR/RT sequences using an extensive array of recombination analysis tools (RIP, SimPlot, bootscan and informative sites), in which ten unique recombinants characterised from seemingly non-epidemiologically linked individuals shared one or more recombination breakpoints with CRF33_01B (CRF33_01B comprised of two subtype B′ fragments recombined with CRF01_AE in the *gag-pol* region [21]). For example, the novel CRF candidate 1 (10MYKJ036, 11MY1RJ704, 11MY1ZK731 and 11MY1EP794) shared one identical breakpoint with CRF33_01B in the *gag* region (**Figure 2A, 2B**). Novel CRF candidate 2 (10MYKJ067, 10MYKJ079 and 04MYKL016) shared the first subtype B′ fragment with CRF33_01B and CRF candidate 3 (10MYKJ016, 10MYKJ052, 11MY1YC672 and 11MY1JJ741) shared both subtype B′ fragments with CRF33_01B with an additional fragment of subtype B′ recombined in the *pol* region. It is important to note that the incorporation of RIP analysis in this study proved to be invaluable in rapidly identifying unique recombinants forms/clades which were otherwise indistinguishable based on the neighbour-joining tree topology, for example in the case of both novel CRF candidates 2 and 3 which were grouped discreetly within the CRF33_01B clade. The high degree of genetic relatedness among the three distinct CRF candidates with CRF33_01B was favourably suggestive of on-going inter-subtype recombination involving CRF33_01B and the co-circulating CRF01_AE and subtype B′ in the country, that may be facilitated by the broad distribution of CRF33_01B among drug injecting networks. Full length genome sequencing of these clades are currently underway to ascertain the complete recombinant profiles for CRF assignment [**50**] followed by phylogenetic inference to discern the possible ancestral relationships among these recombinogenic lineages.

In summary, we describe the first genomic and epidemiological data demonstrating the massive expansion of HIV-1 CRF33_01B among the high-risk injecting drug use population and its subsequent spread into the general population in Malaysia. The discovery of multiple unique recombinant clusters closely related to CRF33_01B also indicated the emergence of a group of novel second-generation CRFs in Southeast Asia. As a result of the dynamics and extreme genetic variance of HIV-1 in the region, continual effort in designing effective treatment and prevention strategies must be improved.

## Supporting Information

Figure S1 **Phylogenetic analysis of the partial** ***gag-*** **PR (HXB2∶1753–2591)** **and partial RT** **(HXB2∶2476–3440) HIV-1 sequences amplified among people who inject drugs (PWID) in Kuala Lumpur, Malaysia.** The partial *gag-*PR (n = 16) and partial RT (n = 14) genes were sequenced, codon-aligned and manually adjusted with the HIV-1 reference subtypes and circulating recombinant forms (CRFs) retrieved from the Los Alamos HIV database (http://www.hiv.lanl.gov/). Neighbour-joining tree was constructed in MEGA 5.05 [34] using Kimura 2-parameter method of nucleotide substitutions to estimate pair-wise evolutionary distance and the reliability of the branching nodes were assessed by bootstrap analysis of 1000 replicates. Reference strains of established and informative HIV-1 genotypes – CRF01_AE, subtype B (including Thai B′), CRF15_01B, CRF34_01B CRF33_01B, CRF48_01B, CRF51_01B, CRF52_01B and other relevant unique recombinant forms (URFs) circulating in Southeast Asia were also included in the analysis to improve tree resolution. Other HIV-1 genotypes – subtypes C, D and SIVcpz reference strains (CPZ.US_MARILYN and CPZ.SIVcpzCAM13) were included as outgroup. The newly emerged unique recombinant clusters identified in this study were highlighted in **Figure S1b** as CRF candidate 1 (10MYKJ036, 11MY1RJ704, 11MY1ZK731 and 11MY1EP794), CRF candidate 2 (10MYKJ067, 10MYKJ079 and 04MYKL016) and CRF candidate 3 (10MYKJ016, 10MYKJ052, 11MY1YC672 and 11MY1JJ741). Bootstrap values of greater than 70% were indicated on the branch nodes. The scale bar represents 5% and 2% genetic distances (in substitutions per site), respectively.
(PDF)

Figure S2 **Bootscan analyses of HIV-1 unique recombinant forms (URFs) constituting three novel clades of circulating recombinant forms (CRFs) isolated from people who inject drugs (PWID) in Malaysia. A**, Schematic representation of the full length recombinant structure of CRF33_01B with the four unique recombination breakpoints labelled as 1–4 (HXB2∶2053–2063 nt, 2375–2416 nt, 2538–2540 nt and 2841–2875 nt, respectively) in the *gag-pol* region [21]. **B**, Bootscanning plots of three novel CRF candidates (1 to 3) and a previously published URF, 06MYKLD46 [45] generated from the 1.6 kb *gag-pol* gene (HXB2∶1753–3440 nt) using SimPlot version 3.5.1 [36]. HIV-1 reference strains CRF01_CM240 (CRF01_AE) and B′_RL42 (Thai subtype B′) were selected as the putative parental genotypes by similarity plotting and C_95IN21068 (subtype C) as an outgroup, with a window size of 200 nucleotides moving along the alignment in increments of 50 nucleotides to define the recombination structures. The shared recombination breakpoints between the URFs were highlighted and in cases where the breakpoints were also shared with CRF33_01B, these breakpoints were further numbered (1 to 4). In comparison with other four isolates of novel CRF candidate 1, 06MYKLD46 displayed distinct recombination structures and breakpoints in the *gag-pol* genes (*as indicated by arrows*) and is thus excluded as a possible candidate.
(EPS)

Figure S3 **Maximum likelihood analysis of HIV-1 CRF33_01B disseminating among various risk groups and geographical origins in Southeast Asia (Malaysia and Indonesia).** The maximum likelihood reconstruction of all available CRF33_01B isolates was estimated by PAUP* v4.0 beta [40]. A total of 242 CRF33_01B sequences (*pol* region, HXB2∶2253–3260) spanning year 2005–2012 among individuals from various risk groups (inclusive of 152 and 90 sequences with known and unknown transmission risks, respectively) and geographical origins (central, northern and eastern states of Malaysia) and two full-length sequences from Indonesia were analyzed. The data set of CRF33_01B_pol_ sequences were amplified from HIV-1 positive samples collected from the following regions: the central states of Malaysia (n = 142) including Kuala Lumpur (n = 54, including four CRF33_01B reference sequences [21], Selangor (n = 86) and Negeri Sembilan (n = 2); the northern states (n = 85) including Kelantan (n = 79) and Perak (n = 6); and the eastern state (n = 13) of Pahang [22]. Indonesian CRF33_01B reference sequences (n = 2) were retrieved from the online database [27] and included in the analyses. Spatial analysis based on phylogenetic reconstruction of CRF33_01B_pol_ data set comprising 242 sequences from various geographical origins revealed vague evidence of directional gene flow from the central to other northern and eastern states, although a high proportion of early transmission events can be traced to PWIDs recruited from the central region.
(EPS)
