# Supplementary material for: Molecular Diversity of HIV-1 among People Who Inject Drugs in Kuala Lumpur, Malaysia: Massive Expansion of Circulating Recombinant Form (CRF) 33_01B and Emergence of Multiple Unique Recombinant Clusters
Source: PLoS One. 2013 May 7;8(5):e62560. doi: 10.1371/journal.pone.0062560 (PMC3646884; doi:10.1371/journal.pone.0062560)

Supplementary Figure 1a (Partial *gag*-PR)

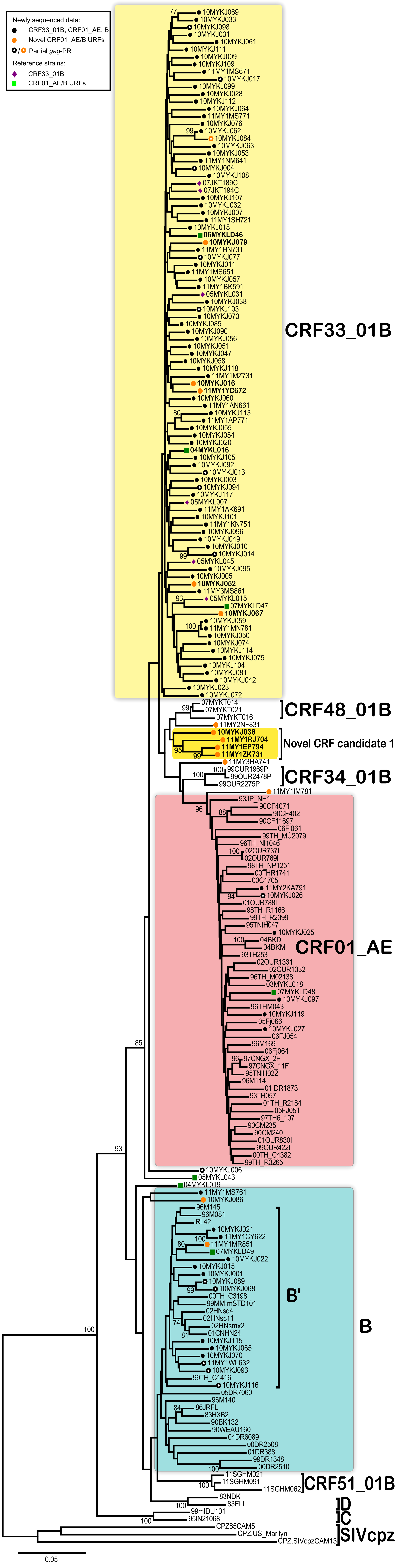

Supplementary Figure 1b (Partial RT)

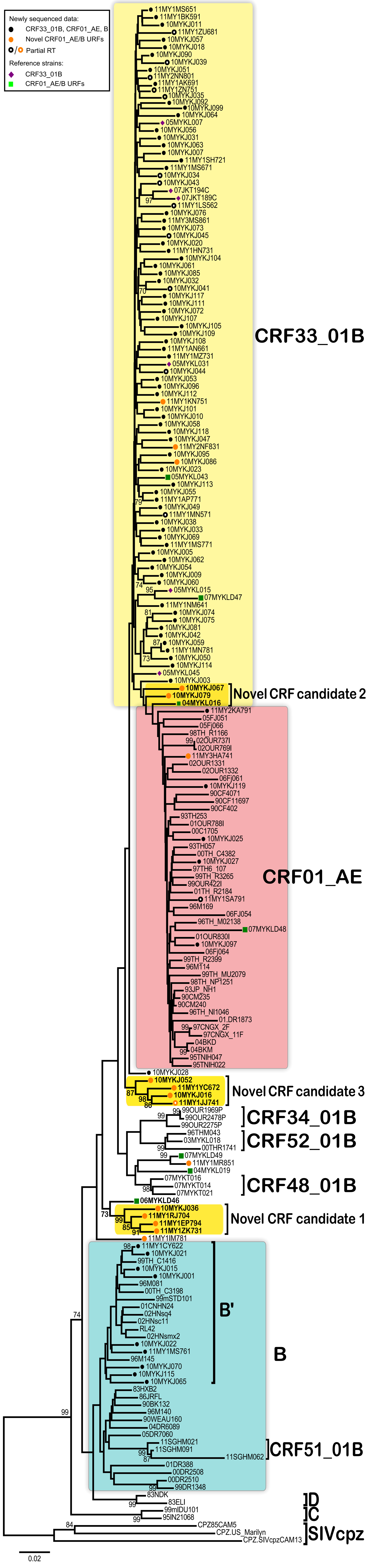

Supplement: Figure S1 — Phylogenetic analysis of the partial gag- PR (HXB2∶1753–2591) and partial RT (HXB2∶2476–3440) HIV-1 sequences amplified among people who inject drugs (PWID) in Kuala Lumpur, Malaysia. The partial gag-PR (n = 16) and partial RT (n = 14) genes were sequenced, codon-aligned and manually adjusted with the HIV-1 reference subtypes and circulating recombinant forms (CRFs) retrieved from the Los Alamos HIV database (http://www.hiv.lanl.gov/). Neighbour-joining tree was constructed in MEGA 5.05 [34] using Kimura 2-parameter method of nucleotide substitutions to estimate pair-wise evolutionary distance and the reliability of the branching nodes were assessed by bootstrap analysis of 1000 replicates. Reference strains of established and informative HIV-1 genotypes – CRF01_AE, subtype B (including Thai B′), CRF15_01B, CRF34_01B CRF33_01B, CRF48_01B, CRF51_01B, CRF52_01B and other relevant unique recombinant forms (URFs) circulating in Southeast Asia were also included in the analysis to improve tree resolution. Other HIV-1 genotypes – subtypes C, D and SIVcpz reference strains (CPZ.US_MARILYN and CPZ.SIVcpzCAM13) were included as outgroup. The newly emerged unique recombinant clusters identified in this study were highlighted in Figure S1b as CRF candidate 1 (10MYKJ036, 11MY1RJ704, 11MY1ZK731 and 11MY1EP794), CRF candidate 2 (10MYKJ067, 10MYKJ079 and 04MYKL016) and CRF candidate 3 (10MYKJ016, 10MYKJ052, 11MY1YC672 and 11MY1JJ741). Bootstrap values of greater than 70% were indicated on the branch nodes. The scale bar represents 5% and 2% genetic distances (in substitutions per site), respectively. (PDF) [file pone.0062560.s001.pdf]
